# Role of *tbc1* in Drosophila embryonic salivary glands

**DOI:** 10.1186/s12860-019-0198-z

**Published:** 2019-06-26

**Authors:** Dorothy M. Johnson, Deborah J. Andrew

**Affiliations:** The Department of Cell Biology, The Johns Hopkins University School of Medicine, 725 N. Wolfe St, Baltimore, MD 21205 USA

**Keywords:** Drosophila, Salivary gland, Apical, Endosome, Golgi, Rab-GAP, Membrane trafficking

## Abstract

**Background:**

*CG4552/tbc1* was identified as a downstream target of Fork head (Fkh), the single Drosophila member of the FoxA family of transcription factors and a major player in salivary gland formation and homeostasis. Tbc1 and its orthologues have been implicated in phagocytosis, the innate immune response, border cell migration, cancer and an autosomal recessive form of non-degenerative Pontocerebellar hypoplasia. Recently, the mammalian Tbc1 orthologue, Tbc1d23, has been shown to bind both the conserved N-terminal domains of two Golgins (Golgin-97 and Golgin-245) and the WASH complex on endosome vesicles. Through this activity, Tbc1d23 has been proposed to link endosomally-derived vesicles to their appropriate target membrane in the trans Golgi (TGN).

**Results:**

In this paper, we provide an initial characterization of Drosophila orthologue, we call *tbc1*. We show that, like its mammalian orthologue, Tbc1 localizes to the trans Golgi. We show that it also colocalizes with a subset of Rabs associated with both early and recycling endosomes. Animals completely missing *tbc1* survive, but females have fertility defects. Consistent with the human disease, loss of *tbc1* reduces optic lobe size and increases response time to mechanical perturbation. Loss and overexpression of *tbc1* in the embryonic salivary glands leads to secretion defects and apical membrane irregularities.

**Conclusions:**

These findings support a role for *tbc1* in endocytic/membrane trafficking, consistent with its activities in other systems.

**Electronic supplementary material:**

The online version of this article (10.1186/s12860-019-0198-z) contains supplementary material, which is available to authorized users.

## Background

Many parts of multicellular organisms are organized as epithelial tubes, including the entire respiratory, circulatory and excretory systems, as well as the digestive tract and secretory organs. Even the central nervous system begins as an epithelial plate, which folds into an epithelial tube before further elaborating into the highly intricate structures of the brain and spinal cord [1]. Epithelial tubes comprise a monolayer of tightly-adherent and polarized cells surrounding a central lumen. In some epithelial organs, there is also an outer layer of supporting cells, such as the myoepithelial cells, which form the layer of smooth muscle that surrounds many secretory organs [2]. All cells in an epithelium have characteristic apical-basal polarity, with the apical surface facing the lumen, which is sometimes filled with an apical extracellular matrix (ECM), and a basal surface contacting a basal ECM, a myoepithelium, or an underlying layer of connective tissue. Epithelial cells are connected to each other and to the cytoskeleton through adherens junctions, tight (vertebrates) or septate (invertebrates) junctions, and desmosomes. Although the basic organization of epithelial tubes is shared, each tubular organ has a unique architecture linked to its primary function. Consider, for example, the morphological differences between the aorta (a simple tube with relatively few side branches), which functions primarily in blood transport, and the lungs (highly-branched anastomosing tubes), which need large surface areas for gas exchange.

The Drosophila embryonic salivary gland (SG) provides an excellent model for studying the specification, formation, and specialization of tubular organs. SGs form in only a few hours and they form in a translucent embryo that develops outside the mother. Although simple in form, the Drosophila embryonic SGs undergo several morphological changes during development. The glands begin as a pair of thickened placodes of around 140 cells each on the ventral surface of the embryo [3]. A subset of cells in a dorsal posterior position of each placode undergo apical constriction and invaginate to form an incipient tube, which continues to elongate as neighboring cells also change shape, rearrange and internalize [4–6]. Once the SG cells contact the overlying gut mesoderm, they turn and migrate posteriorly. The transition from two plates of cells on the surface to fully internalized and correctly positioned tubes is mediated by cell shape changes, cell rearrangement and collective migration, with no cell division or cell death once the cells are specified to form SGs [7]. As the SG cells undergo the morphological events of tube formation, they are also beginning to specialize for their physiological functions. This process includes the amplification of secretory organelles and the initial production of SG specific gene products [8–11].

The salivary gland is specified by the coordinate action of three homeobox-containing transcription factors: Sex combs reduced (Scr), Extradenticle (Exd) and Homothorax (Hth) [12]. These three transcription factors, in turn, activate expression of additional transcription factors, including Fork head (Fkh), which plays a major role in the survival, morphogenesis and specialization of the SG [5, 8, 9, 11, 13]. Among the identified target genes downstream of Fkh activation is a gene originally known as *CG4552* [3]. Based on its homology to vertebrate Tbc1d23, we refer to this gene as *tbc1.*

*tbc1* was independently identified in a large RNAi screen in Drosophila S2 cells, where reduction of *tbc1* was shown to decrease levels of phagocytosis [14]. It was also shown that RNAi knockdown of *tbc1* in the border cells (BC) of the developing Drosophila ovary slows BC migration in one of the two tested lines [15]. A genetic screen in *C. elegans* and in vertebrate macrophages revealed a role for Tbc1 orthologues in the innate immune response [16]. Further studies in the murine immune system revealed that Tbc1d23 attenuates the innate immune response after initiation [17]. Loss of *Tbc1d23* in stimulated macrophages increased both the levels and duration of cytokine production, whereas overexpression of Tbc1d23 did the opposite. A more recent study has shown that Tbc1d23 functions in endosome to Golgi trafficking, linking Golgin-97 and Golgin-245 in the *trans* Golgi to the WASH complex on endosome-derived vesicles [18], revealing a first clear cell biological activity for this protein.

To characterize the role of *tbc1* in the context of epithelial tube formation, we generated a null allele as well as constructs for overexpression of both untagged and tagged versions of Tbc1. We also developed tools to determine the cellular localization of Tbc1 to gain additional insight into its function. Our studies reveal that both loss and overexpression of *tbc1* results in SG secretion defects and irregularities in the lumenal membrane. Also observed with loss of *tbc1* are decreases in the size of the optic lobes of the larval brain and increases in the recovery time following mechanical perturbation.

## Results

**Fig. 1:**
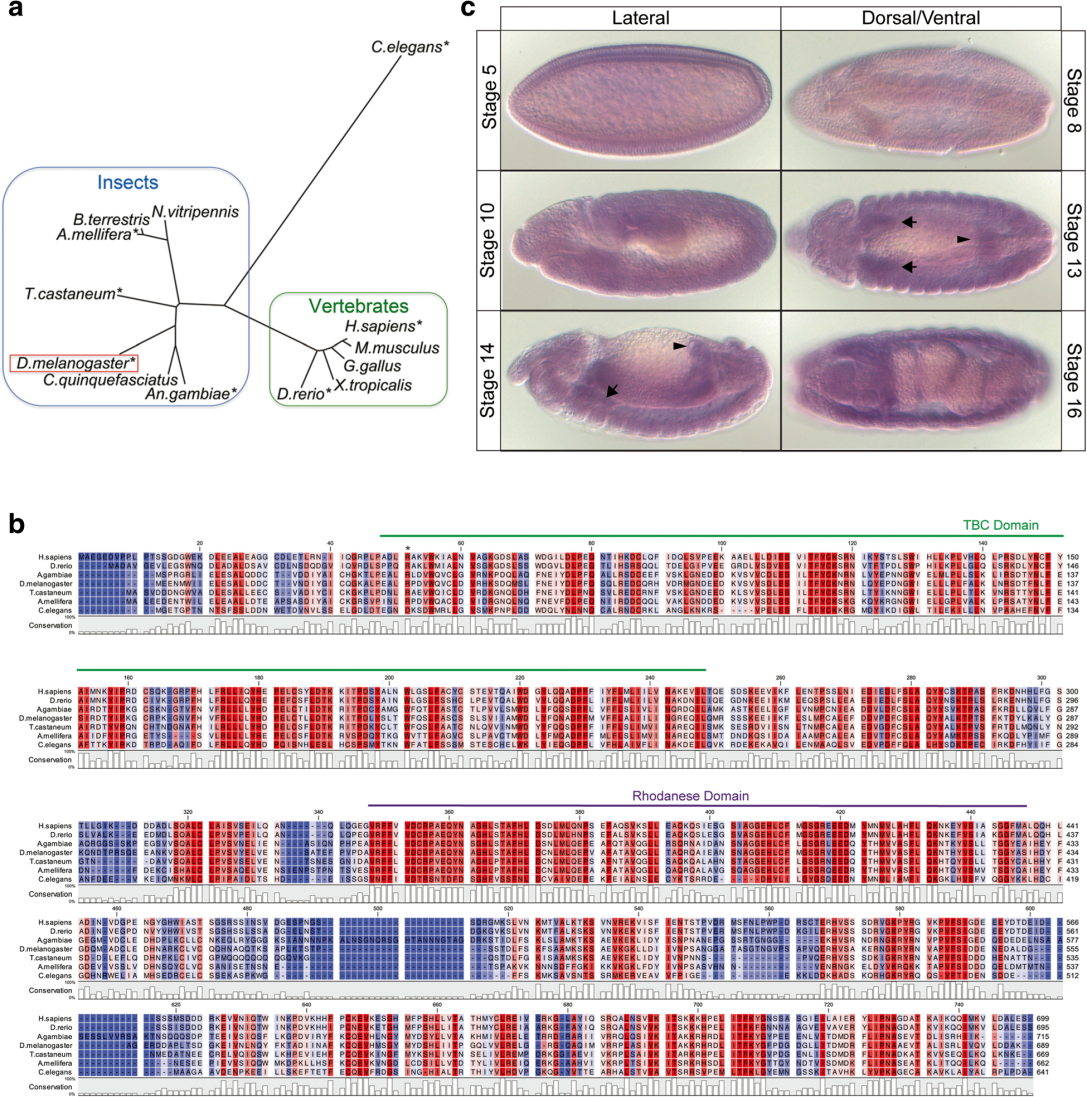
Drosophila tbc1 is highly conserved and is expressed in Drosophila embryos **a** A Phylip unrooted tree analysis of Tbc1 orthologues. Asterisks indicate species included in the alignment. **b** Alignment of a subset the Tbc1 orthologues generated with tools available at the CLC Workbench (Qiagen). Amino acids are colored along a gradient by positional percent conservation across species. Conservation is displayed as a bar chart below the alignment. Blue shading is poor conservation (dark blue 0% conservation), whereas red shading is high conservation (dark red 100% conservation). Geen bar above sequence indicates the TBC domain; purple bar above sequence indicates the Rhodanese-like domain. **c** Whole-mount in situs with probes detecting tbc1 mRNA in wild-type embryos. Black arrows, SG; white arrows, proventriculus; arrowhead, hindgut. In stage 16, there is also hindgut expression that is not visible in the focal plane shown. The Phylip unrooted tree and alignments were constructed with the tools available at [https://www.genome.jp/toolsbin/clustalw]

A BLASTp search with Drosophila Tbc1 identified a single orthologue in each species of higher eukaryotes (Fig. 1a). An alignment of the open reading frames (ORF) from a subset of species revealed that the Tbc1 orthologs contain both a Tre1/Bub-2/Cdc-16p (TBC) domain and a Rhodanese-like domain (Fig. 1). The TBC domain of many of the more distantly related Tbc family members has been shown to have Rab GTPase Activating Protein (GAP) activity, but Tbc1 and its orthologues lack the key sequence motif known to be required for such activity [19]. Thus, it is unclear if Tbc1 acts as a Rab-GAP. The Rhodanese-like domain, has several potential cellular functions, including acting as a phosphatase, an ubiquitin hydrolase, and a sulfur-transferase [20]. Additionally, the presence of the Rhodanese-like domain distinguishes Tbc1 and its orthologues from other TBC-containing proteins. The alignment of Tbc1 family members from several species revealed high conservation throughout the protein, both within and outside of the TBC and Rhodanese-like domains. The existence of only a single highly conserved orthologue in all higher animal species suggests that Tbc1 may have a common function.

In Drosophila, *tbc1* transcripts are maternally contributed [21] and are detectable at low levels in all cells of the developing embryo (Fig. 1c). Zygotic expression of *tbc1* is upregulated in the SG, proventriculus, and hindgut, and the Berkeley Drosophila Genome Project (BDGP) also reports elevated expression in the anterior and posterior midgut primordium [22, 23]. Upregulation in the SG begins early, shortly after the SG is first specified, and continues throughout embryogenesis. *tbc1* expression is also detected at moderate levels in the larval and adult SGs [24, 25]. Because *tbc1* is expressed in the Drosophila SG and is regulated by Fkh, a key factor in SG development and function [3], we investigated the role of *tbc1* in the context of this tissue.

**Fig. 2:**
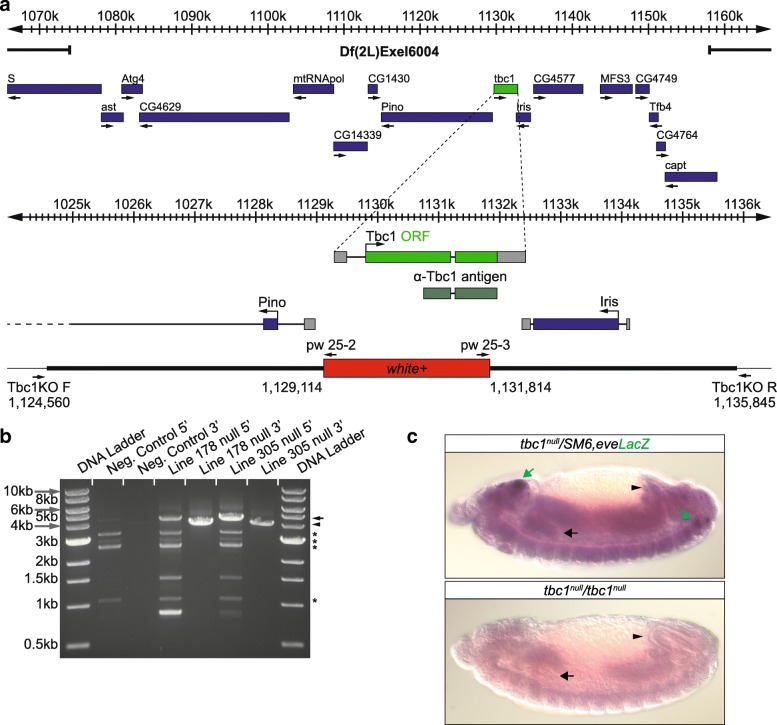
Generation of the *tbc1* null allele. **a** Genomic location of *tbc1*. The deficiency Df(2 L)Exel6004, which deletes *tbc1* and all or part of another 15 genes, is indicated. Small arrows indicate direction of transcription for each of the nearby transcripts. The region removed in the knockout is indicated. *tbc1* KO F and R primers were used with pW25–2 or PW25–3, respectively, to confirm the knockout. The thick black line represents the homologous regions used for generating the knockout. The numbers next to and just below the *white +* insertion indicate exactly which sequences were removed by homologous recombination. **b** PCR of two *tbc1* knockout lines, 178 and 305. Line 178 was used for all the experiments throughout the rest of this work. The negative control for the PCR is one of the lines that is transgenic for the original knockout construct, and maps to a different chromosome. Primers used are those indicated in A. Arrow shows the expected band size for the 5′ end, the arrowhead shows the expected band size for the 3′ end. Asterisks indicate multiple bands also found in the negative control. **c** In situ stains of *tbc1* and *lacZ* mRNA in heterozygous and homozygous *tbc1* knockout siblings. Green arrows indicate *lacZ* expression from the balancer chromosome; Black arrow indicates the salivary gland. *tbc1* mRNA is absent when there is no *lacZ* mRNA (i.e in homozygous knockout embryos)

Existing tools for studying *tbc1* function included a deficiency that removes *tbc1* and an additional 15 genes (Df(2 L)Exel6004) (Fig. 2a), a single P-element insertion line that had no effect on *tbc1* expression (data not shown), and the two RNAi lines that had been tested previously for effects on border cell migration [15]. Since SG expression of both *tbc1* RNAi constructs resulted in high frequency apical membrane irregularities (data not shown), we generated a *tbc1* null allele for further characterizing its SG function. The null allele was made using homologous recombination [26], wherein the entire *tbc1* ORF was replaced with the *white +* eye color marker (Fig. 2a). We also created UAS-*tbc1* transgenic lines to express either untagged or C-terminal GFP-tagged versions of the Tbc1 protein. We made antiserum directed against the C-terminal half of the Tbc1 ORF (Additional file 1: Figure S1). The Tbc1 antiserum detected some weak background staining in *tbc1* null embryos, specifically near the apical surface of SG cells, but it also revealed additional vesicular staining in WT SGs that was not observed in the *tbc1* null SGs (Additional file 1: Figure S1A). A combination of immunohistochemistry (Additional file 1: Figure S1A,B,D) and Westerns of whole embryo extracts (Additional file 1: Figure S1C) indicates that the Tbc1 antiserum recognizes endogenous and overexpressed Tbc1.

**Fig. 3:**
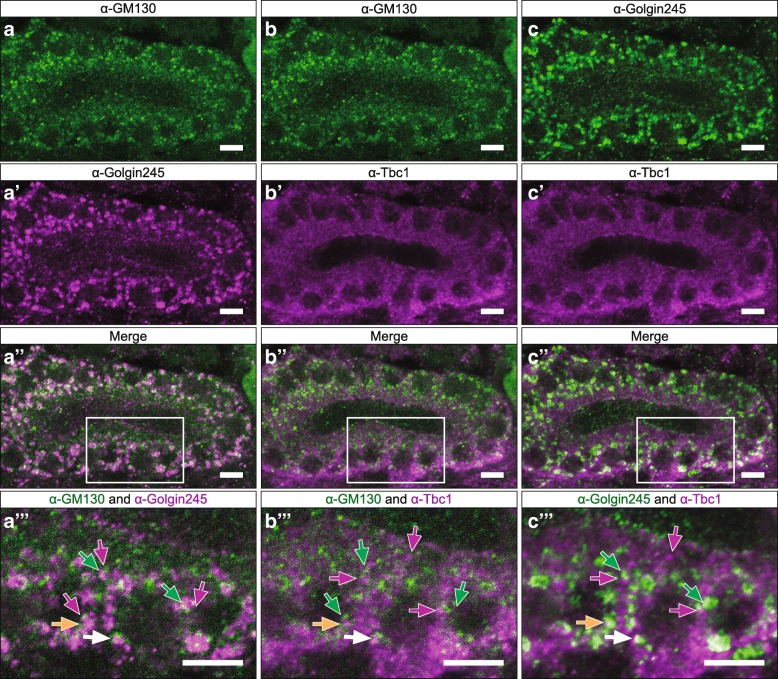
Tbc1 localizes to the trans portion of the *trans* Golgi. A single SG stained for GM130 (cis-Golgi), Tbc1, and Golgin-245 (trans-Golgi). **a** GM130 (geen) and Golgin-245 (magenta) channels. **b** GM130 (green) and Tbc1 (magenta) channels. **c** Golgin-245 (green) and Tbc1 (magenta) channels. Arrows indicate a puncta, white arrow: overlap of all three markers; yellow arrow: overlap of tbc1 and Golgin-245 only. Scale Bar: 5 μm; “higher mag of region indicated in”

To learn where Tbc1 localizes in SG cells, we co-stained SGs expressing Tbc1-GFP with GFP antiserum and antiserum to a variety of other cellular proteins including PH4αSG2 (SG2; an SG-specific ER protein), Cysteine String Protein (CSP; secretory granule marker), and two Golgi proteins. No overlap was observed between Tbc1-GFP and SG2 or between Tbc1-GFP and CSP (Additional file 2: Figure S2). Co-staining with antibodies to a *cis* Golgi marker (GM130) and a *trans* Golgi network (TGN) marker (Golgin-245) revealed that the two Golgi markers do not fully overlap but rather form punctae adjacent to each other (which we call “twin spots”; Fig. 3a). Similarly to Golgin-245, Tbc1 also forms twin spots with GM130 (Fig. 3b). Unlike GM130, Tbc1 partially co-localized with Golgin-245 (Fig. 3c). We frequently observed staining where GM130 punctae would be next to Golgin-245 punctae that, in turn, would either overlap with or be next to Tbc1 punctae. We conclude that Tbc1 localizes to the trans-Golgi network (TGN), with additional Tbc1 staining not associated with the Golgi.

**Fig. 4:**
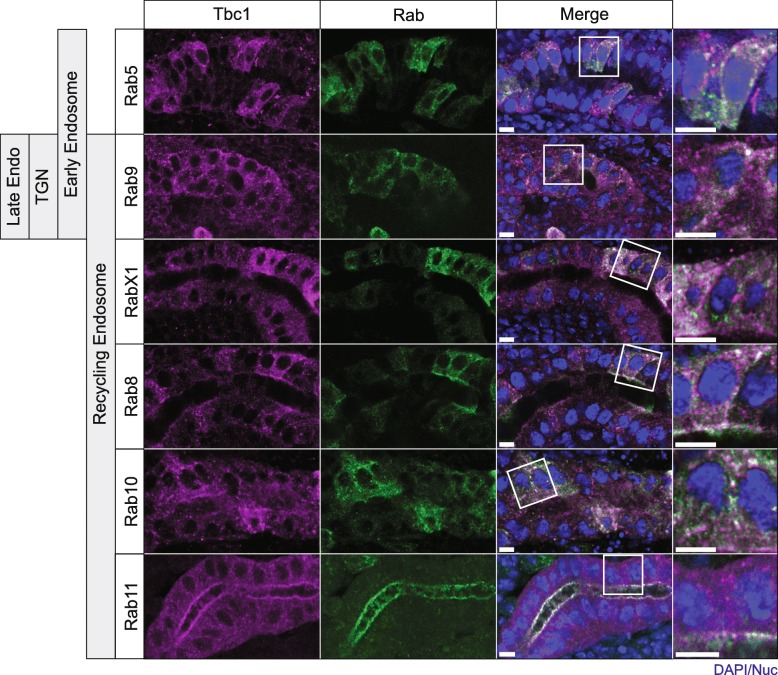
Partial Colocalization of Tbc1 with a subset of Rabs. Tbc1: purple; Rab: Green; DAPI: Blue. UAS-*tbc1* and UAS-YFP-Rab were co-expressed in the SG using a fkh-Gal4 driver on chromosome II that has mosaic expression except for the Rab11 stain (bottom). Anti-Tbc1 was used to detect Tbc1 and anti-GFP was used to detect the Rab proteins in the top five sets of experiments. For Rab11 costaining, endogenous Rab11 was detected with Rab11 antiserum in a fkh-Gal4(III) > UAS-tbc1-GFP background. In this instance, GFP antibody was used to detect Tbc1. Rabs are organized according to Flybase annotations [44]. Scale bar: 5 μm

To gain insight into the additional Tbc1-positive puncta observed in SG cells, we co-expressed untagged Tbc1 and a large set of individual YFP- or GFP-tagged Rab proteins that had been generated by the Scott and Bellen Labs [27]. We used a second chromosome *fkh*-Gal4 line that drives expression in the SG, but to variable levels in different cells. This variability in expression allowed us to focus on protein localization in cells where the relative intensity of the two proteins was both comparable and discernable. We observed partial Tbc1 co-localization with Rab9, RabX1, Rab8, Rab10 and Rab11 (Fig. 4), Rabs that are associated with the recycling endosome. There was also partial co-localization with Rab5, a Rab associated with the early endosome. We observed very little/no overlap in the staining of Tbc1 and the remaining 13 Rabs analyzed in this study (Additional file 3: Figure S3). These Rabs are associated with a wide variety of organelles and cellular structures, including endosomes, ER, Golgi and the plasma membrane.

**Fig. 5:**
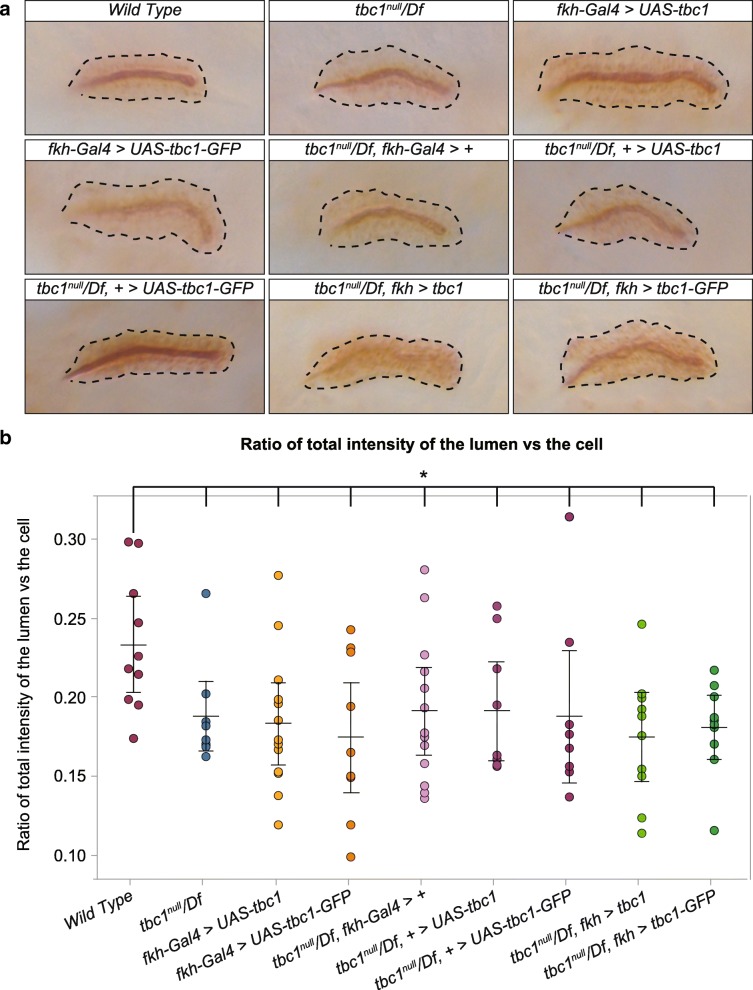
Loss and mis-expression of *tbc1* affects the efficiency of SG secretion. **a** Salivary glands stained for EnP1, an antibody raised to Engrailed that cross-reacts with both secretory vesicles and SG secretions. **b** Ratio of signal intensities across the SG lumens compared to intensities across the cells for the nine different genotypes. For each gland, measurements were taken from 2D images for three separate slices. Note that the wild type sample had the highest average ratio, suggesting more efficient delivery of secretory content to the lumen. The average for WT was significantly different than all other genotypes based on a one tailed unpaired T-Test. * *p*-value< 0.03

The cellular localization of Tbc1 suggested a potential role for this protein in the secretory pathway, shuttling content from the plasma membrane to the trans Golgi, potentially indirectly affecting subsequent trafficking of cargo back to the plasma membrane. To assess whether *tbc1* loss or overexpression has any consequences on secretory function, we used an antiserum generated by the O’Farrell lab that detects an epitope present in SG secretory vesicles and the SG lumen (EnP1; [28]). Since we observed variable levels of EnP1 staining in age-matched embryos even from the same genotypes, we measured the relative intensity of lumenal (secreted) versus cytoplasmic (vesicular) staining in individual stage 15–16 SGs from across all genotypes (Fig. 5). WT SGs had, on average, a significantly higher ratio of lumenal to vesicular EnP1 staining with this marker (23.3%) than observed with either loss of *tbc1* or overexpression of untagged or tagged Tbc1 (17.4–19.1%) (*p* < 0.029 in a one-tailed unpaired T test). Indeed, expression of neither tagged nor untagged Tbc1 in the *tbc1*^*null*^*/Df* SGs restored the WT ratio of lumenal to vesicular EnP1 staining. Overall, this analysis reveals that lumenal cargo is delivered in the absence of Tbc1, but the optimal level of Tbc1 is necessary for the most efficient trafficking of cargo to the lumen.

Preliminary studies had shown that SG expression of *UAS-tbc1* RNAi constructs causes apical membrane irregularities (data not shown). Since *tbc1* mRNA is also provided maternally, we asked if maternal and zygotic versus only zygotic loss of *tbc1* had any differential effects on SG morphology. For this analysis, we obtained embryos from crosses between homozygous *tbc1* null females and homozygous *tbc1* null males, between homozygous *tbc1* null females and males heterozygous for a deficiency that removes *tbc1* (Df(2 L)Exel6004), and between heterozygous *tbc1* females and males heterozygous for the *tbc1* deficiency. We stained these embryos for the apical membrane marker Crb, which nicely outlines the SG lumen, and for βGal, which distinguishes balancer-containing embryos from all other genotypes. From this analysis, we saw no differences in the percentage of SGs with apical surface irregularities between embryos missing both maternal and zygotic *tbc1* versus those missing only zygotic *tbc1* function (data not shown), suggesting that the zygotic supply of Tbc1 is important for apical membrane regularity in the SG.

**Fig. 6:**
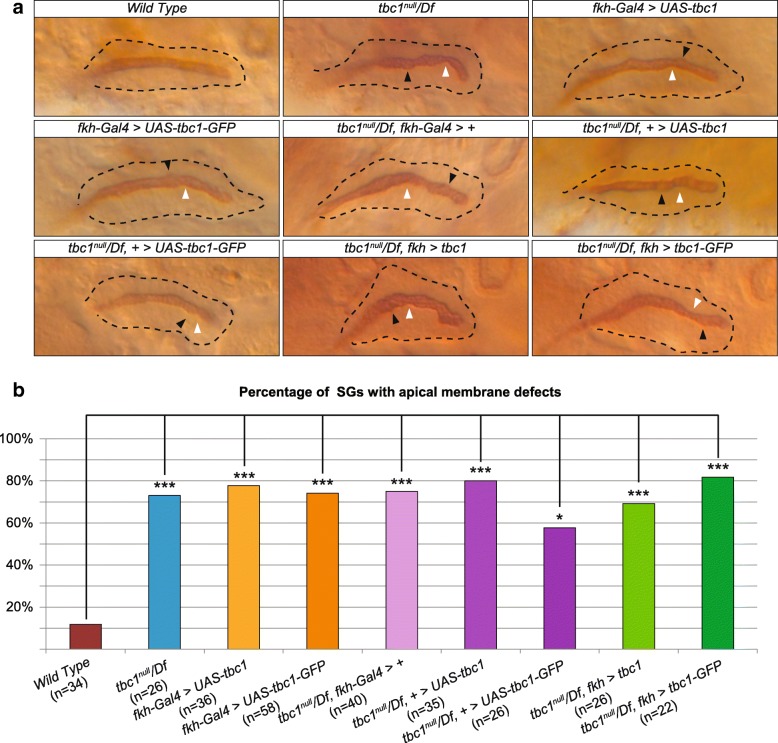
Loss and mis-expression of *tbc1* results in apical membrane irregularities. **a** Examples are shown of SGs from WT embryos and embryos either missing or overexpressing *Tbc1*. Black arrowheads indicate bulges and white arrowheads indicate constrictions. **b** Histogram showing the percentage of SGs with apical membrane irregularities from all of the genotypes shown in C. Wild-type SGs are mostly smooth, whereas all other genotypes show significant increases in apical surface irregularities (Fisher’s test). *: *p*-value = 0.002, ***: *p*-value< 10^− 5^

We next compared SG irregularities in embryos missing *tbc1* function to SGs overexpressing either tagged or untagged versions of *tbc1*. Whereas we observed apical irregularities in only ~ 12% of WT SGs, between 57 and 82% of SGs with different combinations of *tbc1* loss and overexpression had apical irregularities (Fig. 6c,d). Approximately 73% of SGs from trans-heterozygotes of the *tbc1* null and the *tbc1* deficiency had apical membrane defects. Interestingly, 73 to 78% of SGs overexpressing either untagged or tagged *tbc1* in otherwise WT embryos had apical irregularities, indicating that both *tbc1* loss and overexpression causes abnormalities. As expected from the abnormalities associated with Tbc1 overexpression, we did not observe rescue of the loss-of-function defects when we expressed either tagged or untagged *tbc1* in SGs. Consistent with the observations of the luminal cargo staining, this result indicates that there is an optimal level of Tbc1 required for proper regulation of membrane turnover in SG.

Since zygotic loss-of-function mutations in many core secretory pathway components often result in severe defects in the cuticle secreted by the epidermal cells [8, 9, 11], we compared the cuticles of WT and *tbc1* null homozygous first instar larvae (Additional file 4: Figure S4A). Complete maternal and zygotic loss of *tbc1* had no discernable effect on the cuticular structures examined, including the mouthparts (Additional file 4: Figure S4B-D), the denticles (Additional file 4: Figure S4E,F), and the filzkörper (Additional file 4: Figure S4G,H), the small “air filter” tubes filled with cuticular threads and found at the posterior end of the trachea. Indeed, for every aspect of cuticle development we assayed, the *tbc1* null larvae had higher percentage “normal” phenotypes than did our WT Oregon R larvae. We did observe that maternal loss of *tbc1* resulted in only 75% of the embryos secreting a larval cuticle, even when *tbc1* zygotic function was provided paternally (Additional file 5: Figure S5). We saw a similar number of completely undeveloped embryos in our collections for immuno-staining for SG proteins. This finding suggests that the requirement for Tbc1 function in secretion is higher in the SG compared to the developing cuticle.

**Fig. 7:**
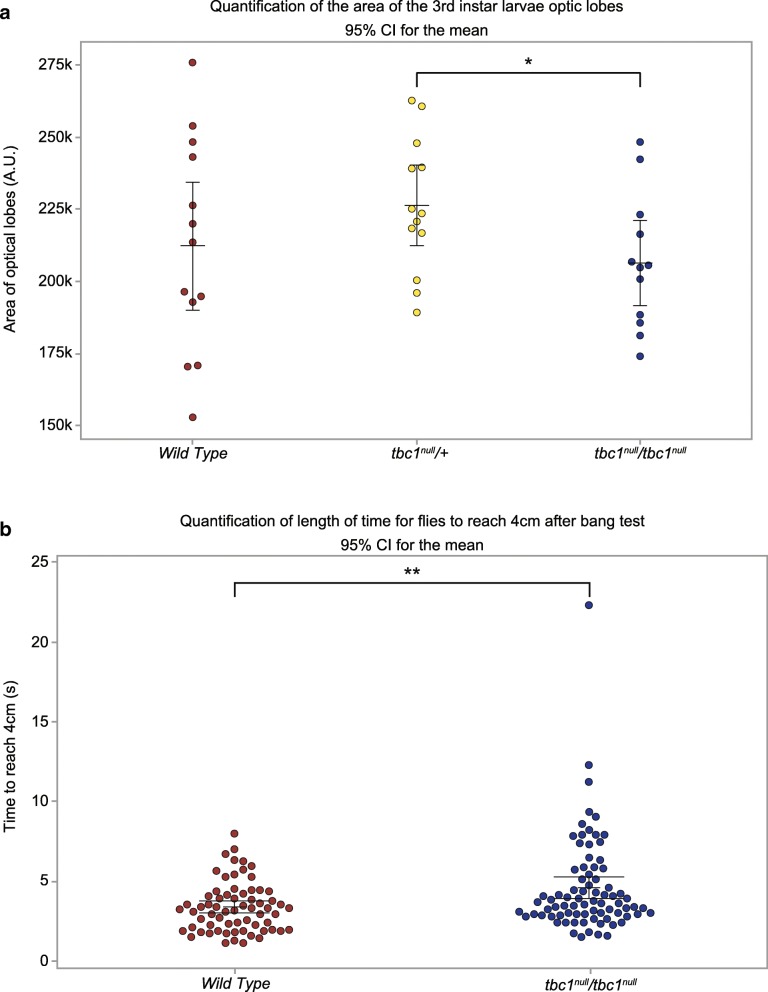
The optic lobes of *tbc1* null larvae are reduced in size and response time following mechanical perturbation is increased (**a**) Optic lobe area from at least 12 samples was determined by outlining the optic lobe in Fiji. **b** The time it took individual flies to travel 4 cm was measured from iPad/Moto g6 movies. For both analyses a one-sided, unpaired T test was the statistical test utilized. *: *p*-value = 0.02. **: *p*-value = 0.002

Because the human disease associated with loss of *TBC1D23* manifests in reduced brain size, we asked if the optic lobes of the brains of fly larvae missing *tbc1* had any irregularities or size differences. The average area of the optic lobes from *tbc1* null larvae was 90% the average area of those isolated from their non-homozygous siblings (Fig. 7a). Although not as significant, the average area of the optic lobes of the *tbc1* homozygous larvae was also reduced compared to wild-type larvae. We also performed a modification of the “bang test” to learn if there were differences in recovery time of flies exposed to mechanical perturbation, which could indicate compromised neuronal function. *tbc1* homozygous adults took an average of 4.584 s to rise a distance of 4 cm, whereas it took wild-type adults an average of 3.3357 s to rise the same distance following four bangs of the vials on a bench top (Fig. 7b).

## Discussion

Here, we show that the Drosophila *tbc1* gene is expressed in all embryonic cells, with elevated levels in the SG, hindgut and proventriculus. We show that the Tbc1 protein is highly conserved among higher eukaryotes and that the Drosophila protein localizes to the trans face of the trans Golgi, consistent with localization of its human orthologue TBC1D23 in HeLa cells and fibroblasts [18, 29]. Our co-staining experiments with tagged versions of the Rabs reveal that Tbc1 also colocalizes with some, but not all, Rab proteins associated with recycling endosomal compartments [27, 30]. Loss of *tbc1* is not lethal; adults homozygous for the null allele are viable and morphologically normal, although the failure of approximately one fourth of embryos from homozygous mothers to develop suggests some reduction in female fertility, a phenotype that could be related to the border cell migration defects previously reported [15]. SGs from null *tbc1* embryos as well as embryos overexpressing *tbc1* are less efficient than WT in the secretion of the cargo protein detected with the EnP1 antiserum. Likewise, SGs from embryos either missing or overexpressing *tbc1* have a high incidence of apical membrane irregularities. Larval cuticles from the *tbc1* null embryos that develop are completely normal; indeed, they show less variation than we observe with our wild-type strain, which has been maintained in the lab more than 25 years and is likely to have picked up a few modifiers that the *Tbc1* mutant lines have not. Larval brain size is reduced and recovery time from mechanical perturbation is increased in *tbc1* homozygotes compared to controls.

The high level of sequence conservation among the Tbc1 orthologues fits with growing evidence for a conserved function for Tbc1 in the innate immune response. Stroschein-Stevenson et al. (2005) demonstrated that *tbc1* knock down decreased the ability of Drosophila S2 cells to phagocytose bacteria (*C. albicans* and *E. coli*), as well as latex beads [14]. *tbc1* was subsequently selected from a genetic screen to identify factors required for innate immunity in both *C. elegans* and in mouse macrophages [16]. Knock down of *tbc1* in *C. elegans* affected expression of several antimicrobial genes (some up and some down) and resulted in diminished survival of worms exposed to pathogenic bacteria. Alper et al., (2008) also demonstrated reduced expression of the cytokine IL6 when *Tbc1d23* was knocked down by shRNA in mouse macrophages [16]. Finally, individuals carrying loss-of-function mutations in the human *TBC1D23* gene are more prone to respiratory infections and sepsis, a phenotype that fits with a role for this gene in innate immunity, but that could alternatively be related to the neurological defects also observed in affected individuals (see below).

Follow-up studies on Tbc1d23 function in innate immunity in mice revealed that it has no role in initiating the response to LPS (a lipopolysaccharide made by gram negative bacteria that functions as a pathogen-associated molecular pattern or PAMP to induce innate immune response), but instead functions in attenuation [17]. Loss of *Tbc1d23* caused increased inflammation as well as notable increases in levels of the cytokines TNFα and IL-6 one to two hours following LPS treatment when compared to controls. Correspondingly, overexpression of *Tbc1d23* in isolated macrophages resulted in diminished mRNA levels for genes encoding a number of cytokines and chemokines implicated in innate immunity (including both TNFα and IL-6) starting one to two hours following LPS induction. Initial induction of innate immunity genes following LPS treatment was not affected by *Tbc1d23* overexpression. Further studies revealed that *Tbc1d23* overexpression inhibited the innate immune response downstream of Toll-like-receptors (TLR) (membrane bound receptors that bind PAMPS) and caused diminished levels of the spliced form of the Xbp1 transcription factor mRNA. Xbp1 mRNA processing, which is normally induced in macrophages in response to LPS and downstream of TLR activation, is required to generate functional Xbp1 protein. In turn, Xbp1 transcriptionally activates expression of innate immunity cytokines, a function independent of its role in the unfolded protein response [31]. Processing of Xbp1 mRNA requires Ire1, an enzyme that sits in the ER membrane and is thought to be activated by the scaffolding proteins downstream of TLR activation. How Tbc1 – a protein proposed to target endosomal vesicles to the trans Golgi [18] – plays into the regulation of Ire1 activity remains unclear, although Tbc1 may have a role in the turnover, regulation or signaling of the TLRs implicated in Xbp1 activation. The endosomal localization of several TLRs, including the ones implicated in Xbp1 activation, is necessary for at least a subset of their signaling activities [32].

*tbc1* expression in the SG (and in other tissues that might be exposed to pathogenic challenges) could be linked to a role in innate immunity. Indeed, both *tbc1* and *Xbp1* are expressed in embryonic SGs starting early in development and continuing at high levels through larval and adult life (D.M.J. and D.J.A., unpubl.; [25]). Both the larval and adult SGs also express at least one effector of innate immunity, known as Drosomycin, although expression of this gene is not detected in the embryonic glands. Irrespective of a potential role in SG innate immunity, our discovery of SG apical membrane irregularities indicates that Tbc1 also plays a role in tissue morphogenesis. As with its role in innate immunity, growing evidence also supports a conserved role for Tbc1 in tissue morphogenesis.

Two independent groups have linked loss-of-function Tbc1 mutations to a non-degenerative form of Pontocerebellar Hypoplasia [29, 33]. One group also demonstrated decreases in hindbrain volume of zebrafish with morphant knockdown of *Tbc1D23*, a phenotype that recapitulates the decreases in volume of both the pons and cerebellum that have been observed in the human patients [29]. The other group used shRNA knockdown of mouse *Tbc1D23* to look for a potential developmental basis for the decreases in brain volume. They showed that there were no changes in cell number or in the number of dividing or dying neurons associated with reduced levels of Tbc1d23, but that *Tbc1d23* knock-down neurons often failed to migrate to the upper cortical layers of the developing brain [33]. This group further demonstrated decreases in the average velocity of retrograde and anterograde transport of NYP-GFP labeled vesicles and of lysosomes (using lysotracker) when *Tbc1d23* was knocked down in fibroblasts. Finally, Ivanova et al. (2017) assayed for morphological changes associated with Tbc1D23 knockdown using Neuro-2 cells induced to differentiate [33]. They saw a significant reduction in the number of cells with a single dominant primary neurite and an increase in the number of cells with many neurites of similar small size. This last finding suggests a role for Tbc1d23 in localizing neurite outgrowth and membrane trafficking. Finding changes in both optic lobe size and in response time following mechanical perturbation in *tbc1* null *Drosophila* hints at similar functions for Tbc1 in fly neuronal differentiation.

## Conclusions

Based on the requirements for *tbc1* function in neurite outgrowth, for the engulfment of bacteria by Drosophila S2 cells, and in the formation of a uniform and smooth apical membrane in Drosophila SGs, we propose that Tbc1 functions to regulate vesicular trafficking for localized membrane expansion. Neurite outgrowth requires delivery of membrane through localized delivery of Golgi-derived vesicles (for review, see [34]). Likewise, bacterial engulfment by phagocytosis requires cells to enwrap bacteria with their own plasma membrane – an event that requires extensive and localized membrane expansion, also mediated by fusion of localized vesicle pools [35]. Indeed, many genes linked to vesicular transport were also identified in the same S2 RNAi screen that linked the loss of *tbc1* to reduced phagocytosis [14]. Finally, elongation of the embryonic SG requires polarized membrane expansion with more growth along the proximal-distal axis than along the circumferential axis of all cells in the elongating tube [36]. Early SG cells have an extensive reservoir of membrane found in microvillar-like structures along the apical surface. This membrane reservoir is depleted by later stages in embryogenesis [28], suggesting that a combination of endocytosis and regulated exocytosis supports the cell elongation associated with tube lengthening. Based on their findings that Tbc1 binds both Golgi proteins and the WASH proteins found on endosomal vesicles, Shin et al. (2017) proposed that Tbc1 functions to increase both the efficiency and specificity of endosome capture to the Golgi compartments [18], a process that, when disrupted, could easily limit the localized growth of membranes dependent upon the pool of available Golgi-derived vesicles. Indeed, similar apical membrane irregularities with loss of *CrebA* function; the transcription factor that upregulates all components of the secretory pathway [10, 37]. Finding that Tbc1 co-localizes with many Rabs associated with endosomal trafficking provides a mechanism for localized subcellular regulation of Tbc1 activity.

Is Tbc1 a Rab-GAP? The signature motifs of bona fide Rab-Gaps are not conserved in this branch of the Tbc1 family [19]. Nonetheless, de Arras et al. (2012) demonstrated that replacement of an Arginine residue (R50A) known to be required for GTPase activity in a closely related Tbc protein resulted in a loss of the Tbc1 overexpression phenotypes observed in macrophages [17]. Interestingly, that residue is not conserved in *C. elegans* Tbc1. Also arguing against a role for Tbc1 as a Rab-GAP is the demonstration by Marin-Valencia et al. (2017) that none of the 55 Rabs they tested showed GTP hydrolysis in the presence of purified Tbc1d23 [29]. Our finding that Drosophila Tbc1 colocalizes with several endosomal Rabs suggests that the TBC homology domain has preserved its ability to bind relevant Rabs; whether it retains an ability to induce GTPase activity remains unclear. The potential Rab interactions observed in the SG could serve the role of localizing Tbc1 activity to limited spatial domains for the dual purposes of supporting development and fighting disease.

## Methods

### Fly Strains

Oregon R was used as the wild-type strain. The *fkh-Gal4* lines were generated previously in the Andrew lab [12]. Several lines were obtained from Bloomington Stock Center (Indianapolis, IN): *w*;*Sco/CyO,ftz-lacZ*, *w*;*TM3/TM6B*, *w;TM3/TM6B, Ubx-lacZ*, and *Sp/CyO,ftz-lacZ;Dr/TM6B,Ubx-lacZ*, y^1^w*;*P{70FLP}11 P{70I-SceI}2B noc*^*Sco*^ */CyO,S*^*2*^. All YFP-Rab lines [27] are noted in Additional file 6: Table S1 and were also obtained from Bloomington Stock Center (Indianapolis, IN). All crosses and collections were performed at 25 °C, unless otherwise noted. Homozygous mutant animals and other genotypes of interest were unambiguously identified based on the absence of *lacZ* expression or α-βgal staining from reporter transgenes inserted on the balancer chromosomes.

### Cloning

The homologous recombination knockout of *tbc1* was performed as described previously [26]. Primers used for the HR amplification and cloning as well as those used for determining if the knockout occurred as originally designed are in Additional file 6: Table S2. The HR PCR-amplified fragments were cloned into the pW25 vector and injected into *w*^*1118*^ embryos by Rainbow Transgenics, LLC (Camarillo, CA). The resulting transgenic insertions were mapped to specific chromosomes by tracking the *white*^*+*^ eye color marker. A line with the insertion on chromosome 3 was then crossed to a line expressing flippase and the restriction enzyme I-Sce1 for excision and linearization of the transgenic construct, as described in [26]. Potential knockout lines were selected for *white*^*+*^ insertions on chromosome 2, where the endogenous gene localizes, and subsequently verified by PCR. See Fly Strains for details on the fly lines used.

UAS-tbc1 and UAS-tbc1 -GFP were cloned using the Gateway Cloning system. The *tbc1* ORF was subcloned from cDNA AT03044 into the pEnterD plasmid using TOPO cloning (Invitrogen). The *tbc1* ORF was then swapped into the pTW or pTWG (Gateway Cloning System; Carnegie Institution) plasmids using LR Recombination (Invitrogen). The pTW construct does not include a protein tag and the pTWG construct adds an in frame GFP ORF to the C-terminus of the *tbc1* ORF. Rainbow Transgenics, LLC injected this construct into *w*^*1118*^ embryos to create the transgenic insertions, which were mapped to specific chromosomes using a combination of chromosome-specific balancer lines. See fly strains.

For bacterial expression of the Tbc1 protein for the production of antiserum, the C-terminal region of the *tbc1* ORF (amino acids 321–689) from cDNA clone AT03044 was subcloned into pET15b using the In-Fusion HD Cloning Kit (Invitrogen; primers in Additional file 6: Table S2).

### Antibody Staining

Embryo fixation and immunohistochemistry were performed as previously described [38]. Normal goat serum (NGS) was used for blocking in immunostainings, except for those that included the goat anti Golgin-245 antiserum, where normal donkey serum (NDS) was used instead. Primary and secondary antibodies, concentrations used, and sources of the antibodies are noted in Additional file 6: Table S3. DAPI staining was done at 2 μg/mL.

### In situ hybridization

In situ hybridization was performed as previously described [39]. Antisense RNA probes were directed to the entire coding region of *tbc1* using the cDNA clone AT03044.

### Antiserum Generation

pET15b-Tbc1 was transformed into BL21-DE3 cells. Induction occurred once the cells reached 0.77 AU with 0.1 M IPTG and cells were grown at 37 °C for another 4 h. Inclusion body preparations to isolate the C-terminal region of Tbc1 followed the procedure described previously [40]. The resulting protein preparation was sent to Covance (Denver, PA) for injection into a rat that had been prescreened for the absence of immuno-reactivity in Drosophila embryos.

### Western Blotting

Western blotting was done as described previously [41]. 10% SDS-PAGE gels were used to size separate protein from whole embryo lysates. The size-separated proteins were then transferred to a polyvinylidene difluoride (PVDF) membrane and blotted as described. Antibodies used, along with concentration and antibody sources, are in Additional file 6: Table S3.

### Microscopy

Confocal microscopy was performed on a Zeiss AxioObserver with 780-Quasar confocal module & FCS (NIH Grant S10 OD016374), a Zeiss AxioObserver with LSM700 confocal module (NIH Grant S10 OD016374), or a Zeiss Axiovert 200 with 510-Meta confocal module. All DIC microscopy was performed with a Zeiss Axiophot 2 with Janoptik ProgResC14 Plus opitical imaging system.

### Tbc1 and secretion efficiency

The ratio of signal intensities across the SG lumens was compared to intensities across the cells. Measurements were taken of three separate slices for each gland and averages were calculated for at least eight samples of each genotype. A one tailed unpaired T-Test was used to determine significance. For these and other quantifications, the primary data and statistical analysis are available upon request.

### Measurements of optic lobe size

3rd instar larval brains were dissected into 1x PBS and stained with DAPI (2 μg/mL) for 10 min and washed with PBS 2x. The brains were mounted with AquaPoly Mount (Polysciences, Inc. Warrington, PA). The brains were imaged with DIC the same day as dissection (microscope described above). Fiji [42] was used to outline the optic lobes and measure the area covered by the tissue, at least 12 examples for all genotypes. A one-sided, unpaired T test was the statistical test utilized to determine significance.

### Measurements of recovery following mechanical perturbation

To assay the climbing ability of adult flies, we modified the climbing assay described by Feany and Bender (2000) [43]. In brief, we collected virgin males and female flies and aged the separately for two days. The flies were incapacitated with CO_2_ and 2–3 individuals were placed into vials and the vials were blinded according to genotype and sex. The flies were allowed to recover for at least 2 h. The vials were individually marked to indicate 4 cm above the food. Using either an iPad or Moto G6 phone, we video recorded tapping the vials three times and allowed the flies to recover for at least 15 s. Occasionally, if not all the flies had fallen to the food, the tapping was repeated after a 15 min (or greater) recovery time. Reviewing the footage, we determined how much time it took for the flies to reach the 4 cm mark after the tapping finished. If the tapping had to be repeated, we only used the second set. Additionally, any flies that never reached the 4 cm mark or were not in focus when they passed the 4 cm mark were not included in the analysis (less than 15 individuals). A one-sided, unpaired T test was the statistical test utilized to determine if differences between genotypes were significant.

## Additional files

Additional file 1: **Figure S1.** Development of Tbc1 antiserum and testing of transgenic lines. A) Tbc1 antiserum staining in wild type and *tbc1*^*Null*^ SGs detected by immunofluorescence. B) HRP staining of wildtype embryos and embryos with SG overexpression (using the *fkh-Gal4* driver) of UAS-untagged and UAS-GFP tagged *tbc1* using Tbc1 antiserum. SG is outlined with a dashed line. C) Western blotting for Tbc1 (top), GFP (middle) and βtub (bottom). Full length, untagged tbc1: 77.5 kDa predicted molecular weight; Full length, GFP tagged: 106.7 kDa predicted molecular weight. Wild type: OR; Untagged: tub-Gal4 > UAS-tbc1; Tagged: tub-Gal4 > UAS-tbc1-GFP. Black arrowhead: Untagged Tbc1 size; Green arrowhead: expected size of GFP tagged Tbc1; *: non-specific bands. D) Tbc1 (magenta) and GFP (green) staining of untagged (top) and GFP-tagged Tbc1 expressed in the SG using *fkh-Gal4*. GFP and Tbc1 staining fully overlap in salivary glands expressing Tbc1 -GFP. Blue: DAPI. Scale Bar: 5 μm. (PDF 3979 kb)

Additional file 2: **Figure S2.** Tbc1 does not co-localize with SG2 or CSP. UAS-*tbc1*-GFP driven by *fkh*-Gal4 and immuno-stained for GFP (magenta). A) Costaining with the ER marker SG2 (green). B) Costaining with the secretory vesicle marker dCSP1 (green). Blue: DAPI. Scale Bar: 5 μm. (PDF 1493 kb)

Additional file 3: **Figure S3.** Tbc1 does not colocalize with a subset of Rabs. Tbc1: magenta; YFP-Rab or GFP-Rab: Green; DAPI: Blue. UAS-Tbc1 and UAS-YFP-Rab (or, in the case of Rab7, UAS-GFP-Rab7) were expressed using a *fkh-Gal4* driver on chromosome II that has mosaic SG expression. *: areas where Tbc1 staining is too intense to discern if overlap exists. Scale Bar: 5 μm. (PDF 7527 kb)

Additional file 4: **Figure S4.** Loss of *tbc1* does not affect larval cuticle morphology. (A) 100X dark field images of lateral views of cuticle preparations from wild type and *tbc1* null homozygotes. (B) 400X phase images of the cuticle preparations showing the mouthparts of a wild type and *tbc1* null homozygotes larva. (C,D) Quantification of irregularities and pigmentation defects found in wild type and *tbc1* null mouthparts. (E) 400X images of the A4 denticle belts from wild type and *tbc1* null larvae. (F) Quantification of largest denticle size in WT and *tbc1* null larvae. (G) 400X images of filzkörper (FK) from wild type and *tbc1* null larvae. (H) Quantification of structural FK irregularities in wild type and *tbc1* null larvae. (PDF 8359 kb)

Additional file 5: **Figure S5.** Maternal loss of *tbc1* increases the percentage of progeny that completely fail to produce cuticle Wild-type (Oregon R) or *tbc1* homozygous females were crossed to either wild-type or *tbc1* homozygous males. Between 85 and 92% of progeny of WT females developed into first instar larvae, whereas only between 65 and 75% of progeny of *tbc1* null females developed into first instar larvae. (PDF 116 kb)

Additional file 6: **Table S1.** Rab-YFP lines used. **Table S2.** Primers used. **Table S3** Antibodies used (DOCX 98 kb)

## Abbreviations

Df: Deficiency
ER: Endoplasmic reticulum
Exd: Extradenticle
Fkh: Fork head
GAP: GTPase Activating Protein
GFP: Green fluorescent protein
hth: Homothorax
IL-6: Interleukin 6
IRE-1: Inositol-requiring enzyme 1
ORF: open reading frame
PAMP: Pathogen-associated molecular pattern
RabGAP: Rab GTPase Activating Protein
RNAi: RNA interference
Scr: Sex combs reduced
SG: Salivary gland
shRNA: Short Hairpin RNA
TBC: Tre1/Bub-2/Cdc-16p
TGN: Trans Golgi network
TLR: Toll-like receptor
TNFα: Tumor necrosis factor alpha
UAS: Upstream activating sequence
WT: Wild type
Xbp1: X-box binding protein 1
YFP: Yellow fluorescent protein

## References

[1] Colas JF, Schoenwolf GC (2001). Towards a cellular and molecular understanding of neurulation. Dev Dyn.

[2] Andrew DJ, Ewald AJ (2010). Morphogenesis of epithelial tubes: Insights into tube formation, elongation, and elaboration. Dev Biol.

[3] Maruyama R, Andrew DJ (2012). Drosophila as a model for epithelial tube formation. Dev Dyn.

[4] Myat MM, Andrew DJ (2000). Organ shape in the Drosophila salivary gland is controlled by regulated, sequential internalization of the primordia. Development.

[5] Chung S, Kim S, Andrew DJ. Uncoupling apical constriction from tissue invagination. Elife. 2017;6. 10.7554/eLife.22235. 10.7554/eLife.22235 PMC5338918 28263180

[6] Sanchez-Corrales YE, Blanchard GB, Röper K. Radially patterned cell behaviours during tube budding from an epithelium. Elife. 2018;7. 10.7554/eLife.35717. 10.7554/eLife.35717 PMC6089598 30015616

[7] Chung S, Hanlon CD, Andrew DJ (2014). Building and specializing epithelial tubular organs: the Drosophila salivary gland as a model system for revealing how epithelial organs are specified, form and specialize. Wiley Interdiscip Rev Dev Biol.

[8] Abrams EW, Andrew DJ (2005). CrebA regulates secretory activity in the Drosophila salivary gland and epidermis. Development.

[9] Abrams EW, Mihoulides WK, Andrew DJ (2006). Fork head and Sage maintain a uniform and patent salivary gland lumen through regulation of two downstream target genes, PH4alphaSG1 and PH4alphaSG2. Development.

[10] Fox RM, Hanlon CD, Andrew DJ (2010). The CrebA/Creb3-like transcription factors are major and direct regulators of secretory capacity. J Cell Biol.

[11] Fox RM, Vaishnavi A, Maruyama R, Andrew DJ (2013). Organ-specific gene expression: the bHLH protein Sage provides tissue specificity to Drosophila FoxA. Development.

[12] Henderson KD, Andrew DJ (2000). Regulation and function of Scr, exd, and hth in the Drosophila salivary gland. Dev Biol.

[13] Myat MM, Andrew DJ (2000). Fork head prevents apoptosis and promotes cell shape change during formation of the Drosophila salivary glands. Development.

[14] Stroschein-Stevenson SL, Foley E, O'Farrell PH, Johnson AD (2006). Identification of Drosophila gene products required for phagocytosis of Candida albicans. PLoS Biol.

[15] Laflamme C, Assaker G, Ramel D, Dorn JF, She D, Maddox PS, Emery G (2012). Evi5 promotes collective cell migration through its Rab-GAP activity. J Cell Biol.

[16] Alper S, Laws R, Lackford B, Boyd WA, Dunlap P, Freedman JH, Schwartz DA (2008). Identification of innate immunity genes and pathways using a comparative genomics approach. Proc Natl Acad Sci U S A.

[17] De Arras L, Yang IV, Lackford B, Riches DW, Prekeris R, Freedman JH, Schwartz DA, Alper S (2012). Spatiotemporal inhibition of innate immunity signaling by the Tbc1d23 RAB-GAP. J Immunol.

[18] Shin JJH, Gillingham AK, Begum F, Chadwick J, Munro S (2017). TBC1D23 is a bridging factor for endosomal vesicle capture by golgins at the trans-Golgi. Nat Cell Biol.

[19] Gabernet-Castello C, O'Reilly AJ, Dacks JB, Field MC (2013). Evolution of Tre-2/Bub2/Cdc16 (TBC) Rab GTPase-activating proteins. Mol Biol Cell.

[20] Cipollone R, Ascenzi P, Visca P (2007). Common themes and variations in the rhodanese superfamily. IUBMB Life.

[21] Graveley BR, Brooks AN, Carlson JW, Duff MO, Landolin JM, Yang L, Artieri CG, van Baren MJ, Boley N, Booth BW (2011). The developmental transcriptome of Drosophila melanogaster. Nature.

[22] Tomancak P, Beaton A, Weiszmann R, Kwan E, Shu S, Lewis SE, Richards S, Ashburner M, Hartenstein V, Celniker SE (2002). Systematic determination of patterns of gene expression during Drosophila embryogenesis. Genome Biol.

[23] Tomancak P, Berman BP, Beaton A, Weiszmann R, Kwan E, Hartenstein V, Celniker SE, Rubin GM (2007). Global analysis of patterns of gene expression during Drosophila embryogenesis. Genome Biol.

[24] Chintapalli Venkateswara R, Wang Jing, Dow Julian A T (2007). Using FlyAtlas to identify better Drosophila melanogaster models of human disease. Nature Genetics.

[25] Thurmond Jim, Goodman Joshua L, Strelets Victor B, Attrill Helen, Gramates L Sian, Marygold Steven J, Matthews Beverley B, Millburn Gillian, Antonazzo Giulia, Trovisco Vitor, Kaufman Thomas C, Calvi Brian R, Perrimon Norbert, Gelbart Susan Russo, Agapite Julie, Broll Kris, Crosby Lynn, Santos Gilberto dos, Emmert David, Gramates L. Sian, Falls Kathleen, Jenkins Victoria, Matthews Beverley, Sutherland Carol, Tabone Christopher, Zhou Pinglei, Zytkovicz Mark, Brown Nick, Antonazzo Giulia, Attrill Helen, Garapati Phani, Holmes Alex, Larkin Aoife, Marygold Steven, Millburn Gillian, Pilgrim Clare, Trovisco Vitor, Urbano Pepe, Kaufman Thomas, Calvi Brian, Czoch Bryon, Goodman Josh, Strelets Victor, Thurmond Jim, Cripps Richard, Baker Phillip (2018). FlyBase 2.0: the next generation. Nucleic Acids Research.

[26] Gong W. J., Golic K. G. (2003). Ends-out, or replacement, gene targeting in Drosophila. Proceedings of the National Academy of Sciences.

[27] Zhang Jun, Schulze Karen L., Hiesinger P. Robin, Suyama Kaye, Wang Stream, Fish Matthew, Acar Melih, Hoskins Roger A., Bellen Hugo J., Scott Matthew P. (2007). Thirty-One Flavors of Drosophila Rab Proteins. Genetics.

[28] Myat Monn Monn, Andrew Deborah J. (2002). Epithelial Tube Morphology Is Determined by the Polarized Growth and Delivery of Apical Membrane. Cell.

[29] Marin-Valencia Isaac, Gerondopoulos Andreas, Zaki Maha S., Ben-Omran Tawfeg, Almureikhi Mariam, Demir Ercan, Guemez-Gamboa Alicia, Gregor Anne, Issa Mahmoud Y., Appelhof Bart, Roosing Susanne, Musaev Damir, Rosti Basak, Wirth Sara, Stanley Valentina, Baas Frank, Barr Francis A., Gleeson Joseph G. (2017). Homozygous Mutations in TBC1D23 Lead to a Non-degenerative Form of Pontocerebellar Hypoplasia. The American Journal of Human Genetics.

[30] Bhuin Tanmay, Roy Jagat Kumar (2014). Rab proteins: The key regulators of intracellular vesicle transport. Experimental Cell Research.

[31] Martinon Fabio, Chen Xi, Lee Ann-Hwee, Glimcher Laurie H (2010). TLR activation of the transcription factor XBP1 regulates innate immune responses in macrophages. Nature Immunology.

[32] Rosadini Charles V, Kagan Jonathan C (2017). Early innate immune responses to bacterial LPS. Current Opinion in Immunology.

[33] Ivanova Ekaterina L., Mau-Them Frédéric Tran, Riazuddin Saima, Kahrizi Kimia, Laugel Vincent, Schaefer Elise, de Saint Martin Anne, Runge Karen, Iqbal Zafar, Spitz Marie-Aude, Laura Mary, Drouot Nathalie, Gérard Bénédicte, Deleuze Jean-François, de Brouwer Arjan P.M., Razzaq Attia, Dollfus Hélène, Assir Muhammad Zaman, Nitchké Patrick, Hinckelmann Maria-Victoria, Ropers Hilger, Riazuddin Sheikh, Najmabadi Hossein, van Bokhoven Hans, Chelly Jamel (2017). Homozygous Truncating Variants in TBC1D23 Cause Pontocerebellar Hypoplasia and Alter Cortical Development. The American Journal of Human Genetics.

[34] Pfenninger Karl H. (2009). Plasma membrane expansion: a neuron's Herculean task. Nature Reviews Neuroscience.

[35] Swanson Joel A. (2008). Shaping cups into phagosomes and macropinosomes. Nature Reviews Molecular Cell Biology.

[36] Pirraglia C., Walters J., Myat M. M. (2010). Pak1 control of E-cadherin endocytosis regulates salivary gland lumen size and shape. Development.

[37] Andrew DJ, Baig A, Bhanot P, Smolik SM, Henderson KD. The Drosophila dCREB-A gene is required for dorsal/ventral patterning of the larval cuticle. Development. 1997;124(1):181–193. 10.1242/dev.124.1.181 9006079

[38] Reuter R, Scott MP. Expression and function of the homoeotic genes Antennapedia and Sex combs reduced in the embryonic midgut of Drosophila. Development. 1990;109(2):289–303. 10.1242/dev.109.2.289 1976087

[39] Lehmann R, Tautz D. In situ hybridization to RNA. Methods Cell Biol. 1994;44:575–598. 10.1016/s0091-679x(08)60933-4 7535885

[40] Hanlon CD, Andrew DJ. Drosophila FoxL1 non-autonomously coordinates organ placement during embryonic development. Dev Biol. 2016;419(2):273–284. 10.1016/j.ydbio.2016.09.007 PMC5237845 27618755

[41] Ismat A, Cheshire AM, Andrew DJ (2013). The secreted AdamTS-A metalloprotease is required for collective cell migration. Development.

[42] Schindelin J, Arganda-Carreras I, Frise E, Kaynig V, Longair M, Pietzsch T, Preibisch S, Rueden C, Saalfeld S, Schmid B (2012). Fiji: an open-source platform for biological-image analysis. Nat Methods.

[43] Feany MB, Bender WW (2000). A Drosophila model of Parkinson's disease. Nature.

[44] Gaudet P, Livstone MS, Lewis SE, Thomas PD (2011). Phylogenetic-based propagation of functional annotations within the Gene Ontology consortium. Brief Bioinform.

