# Supplementary figures and images for: Role of tbc1 in Drosophila embryonic salivary glands
Source: BMC Mol Cell Biol. 2019 Jun 26;20:19. doi: 10.1186/s12860-019-0198-z (PMC6595604; doi:10.1186/s12860-019-0198-z)

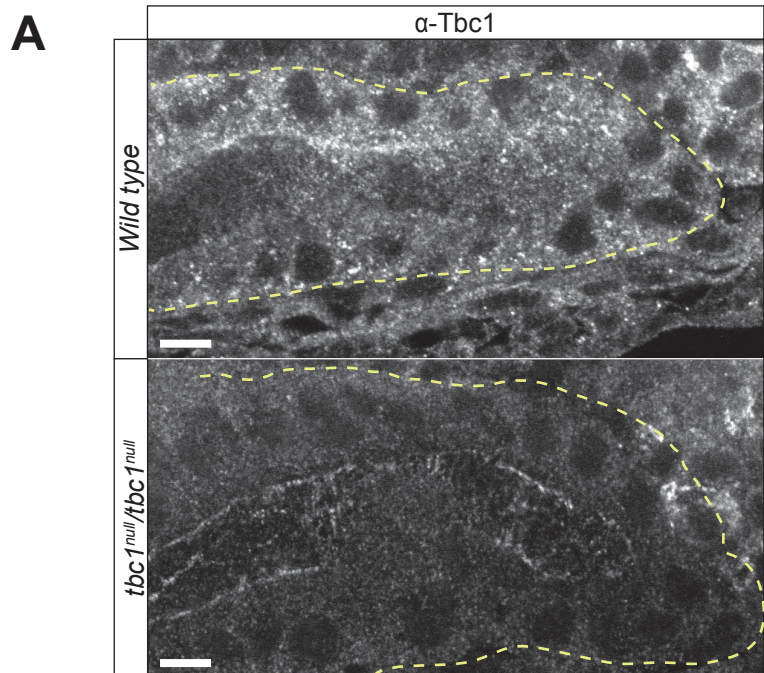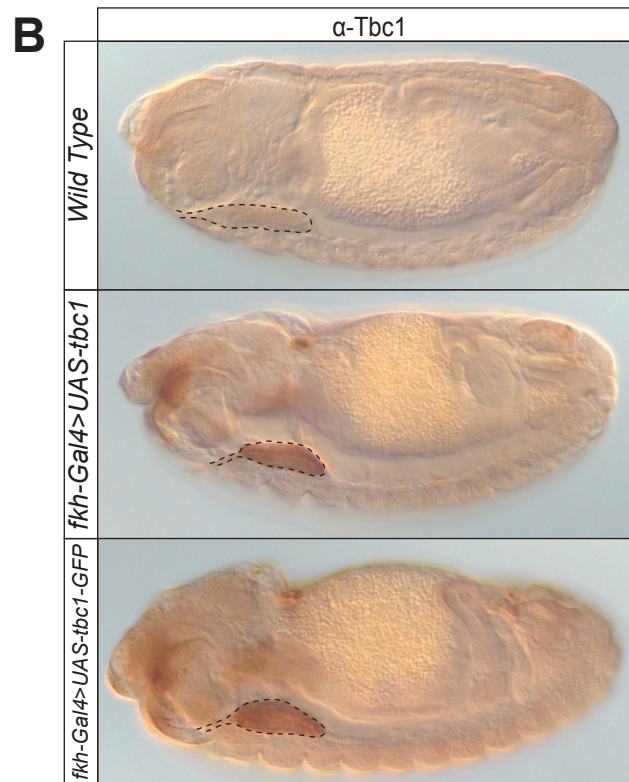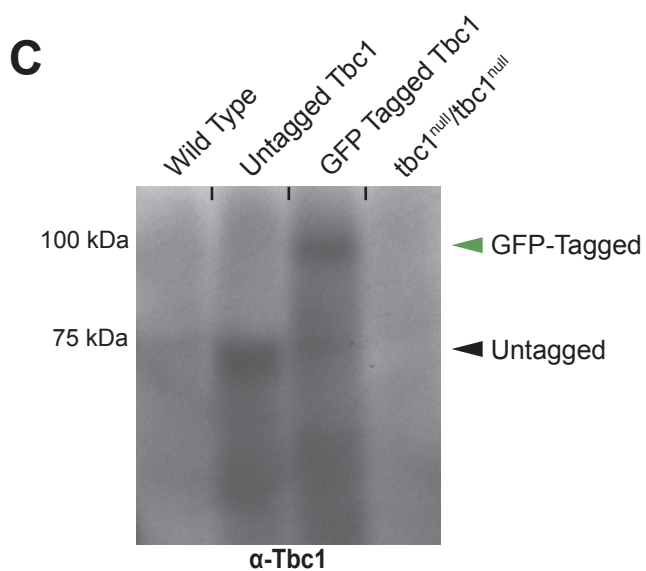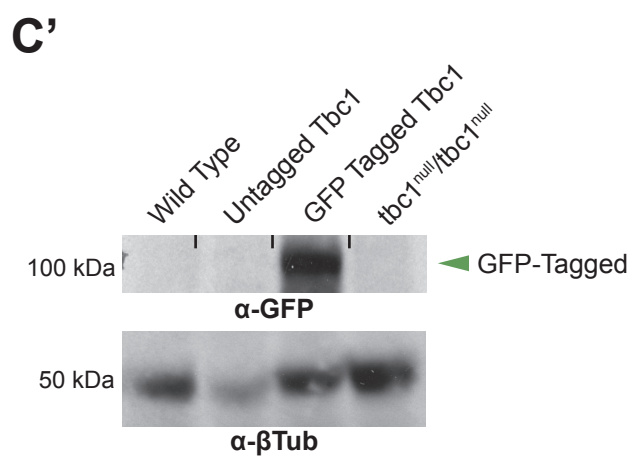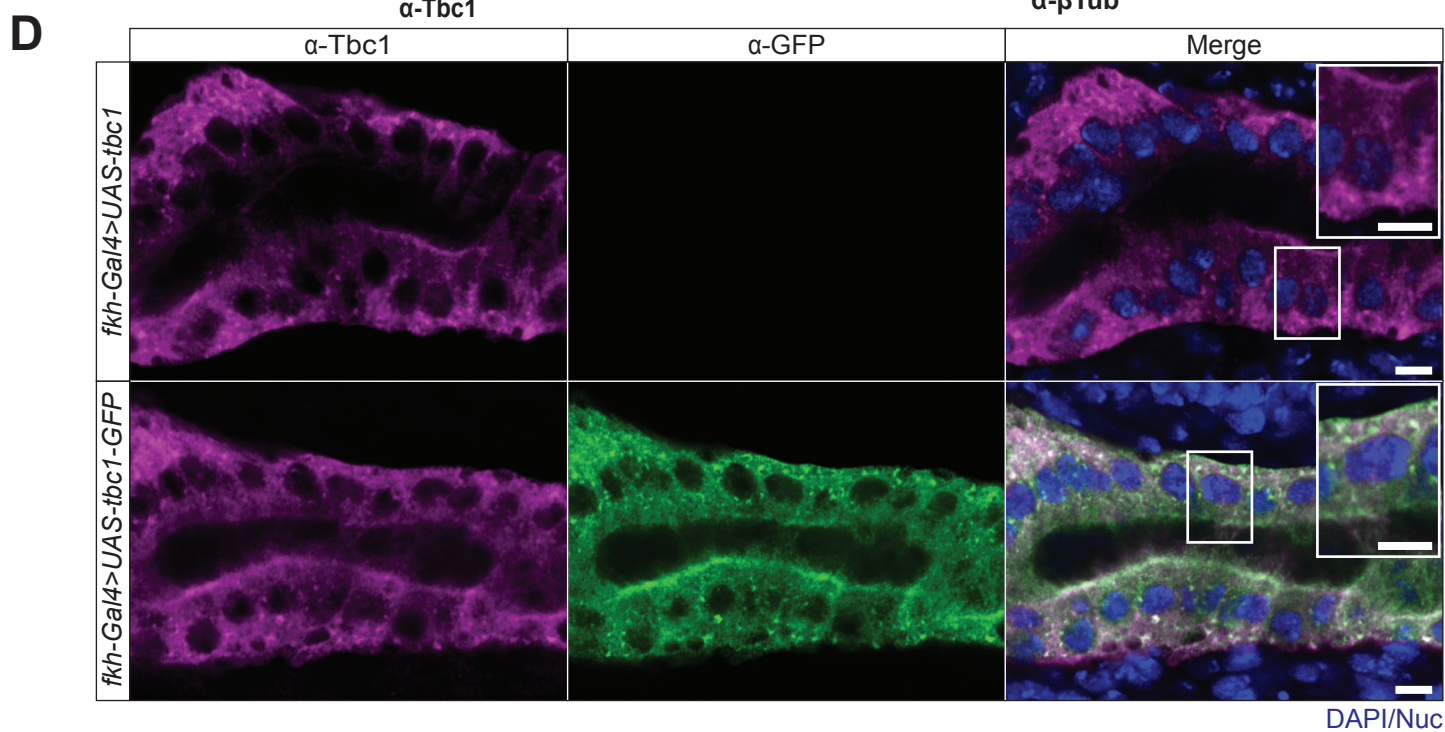

Supplement: Supplementary file 1 — Figure S1. Development of Tbc1 antiserum and testing of transgenic lines. A) Tbc1 antiserum staining in wild type and tbc1Null SGs detected by immunofluorescence. B) HRP staining of wildtype embryos and embryos with SG overexpression (using the fkh-Gal4 driver) of UAS-untagged and UAS-GFP tagged tbc1 using Tbc1 antiserum. SG is outlined with a dashed line. C) Western blotting for Tbc1 (top), GFP (middle) and βtub (bottom). Full length, untagged tbc1: 77.5 kDa predicted molecular weight; Full length, GFP tagged: 106.7 kDa predicted molecular weight. Wild type: OR; Untagged: tub-Gal4 > UAS-tbc1; Tagged: tub-Gal4 > UAS-tbc1-GFP. Black arrowhead: Untagged Tbc1 size; Green arrowhead: expected size of GFP tagged Tbc1; *: non-specific bands. D) Tbc1 (magenta) and GFP (green) staining of untagged (top) and GFP-tagged Tbc1 expressed in the SG using fkh-Gal4. GFP and Tbc1 staining fully overlap in salivary glands expressing Tbc1 -GFP. Blue: DAPI. Scale Bar: 5 μm. (PDF 3979 kb) [file 12860_2019_198_MOESM1_ESM.pdf]

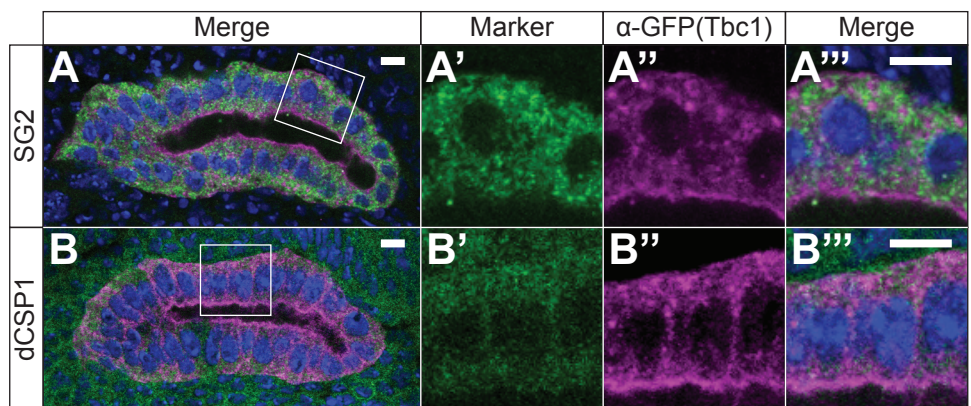

DAPI/Nuc

Supplement: Supplementary file 2 — Figure S2. Tbc1 does not co-localize with SG2 or CSP. UAS-tbc1-GFP driven by fkh-Gal4 and immuno-stained for GFP (magenta). A) Costaining with the ER marker SG2 (green). B) Costaining with the secretory vesicle marker dCSP1 (green). Blue: DAPI. Scale Bar: 5 μm. (PDF 1493 kb) [file 12860_2019_198_MOESM2_ESM.pdf]

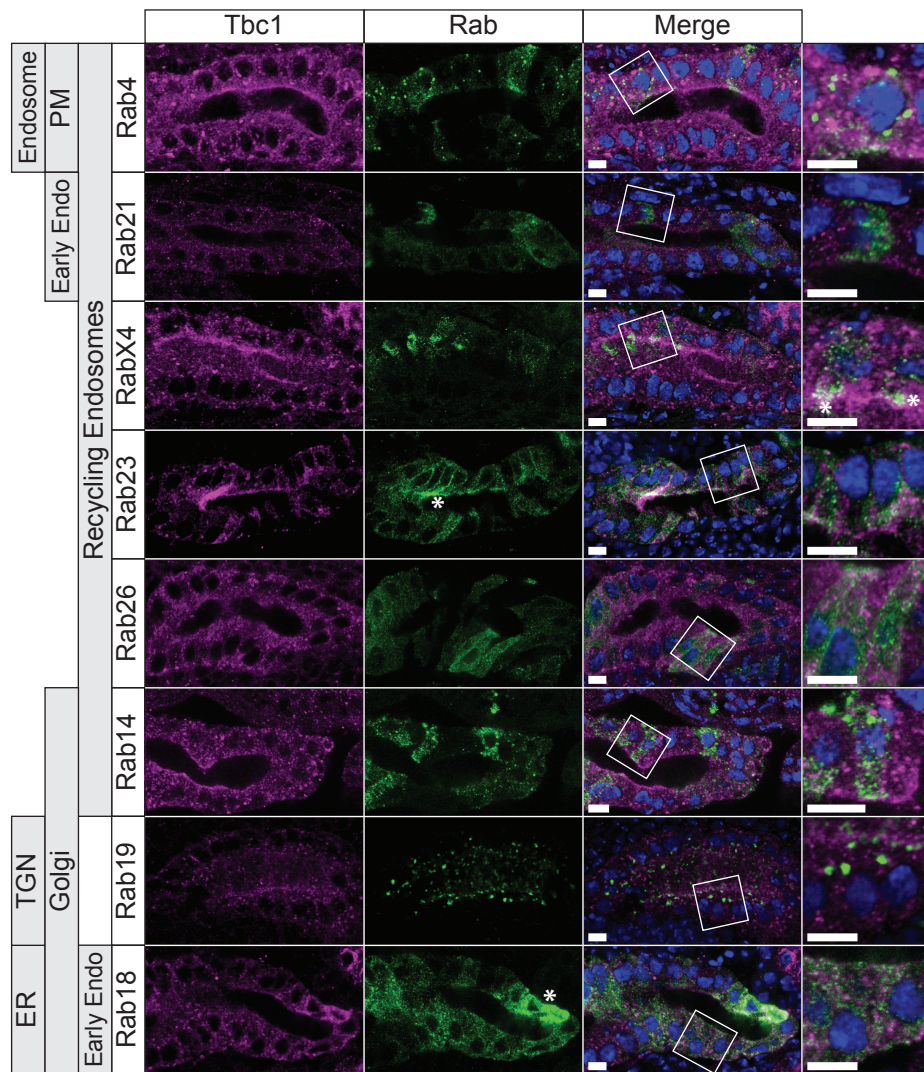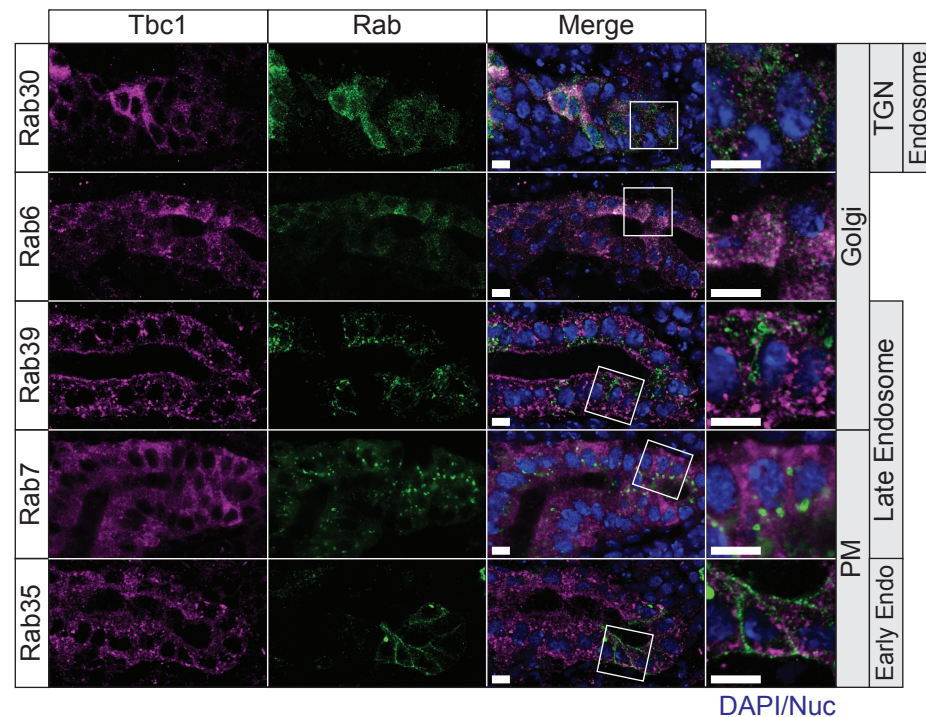

Supplement: Supplementary file 3 — Figure S3. Tbc1 does not colocalize with a subset of Rabs. Tbc1: magenta; YFP-Rab or GFP-Rab: Green; DAPI: Blue. UAS-Tbc1 and UAS-YFP-Rab (or, in the case of Rab7, UAS-GFP-Rab7) were expressed using a fkh-Gal4 driver on chromosome II that has mosaic SG expression. *: areas where Tbc1 staining is too intense to discern if overlap exists. Scale Bar: 5 μm. (PDF 7527 kb) [file 12860_2019_198_MOESM3_ESM.pdf]

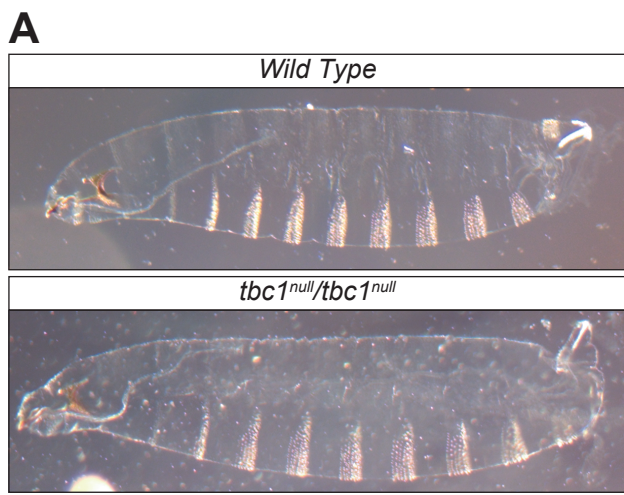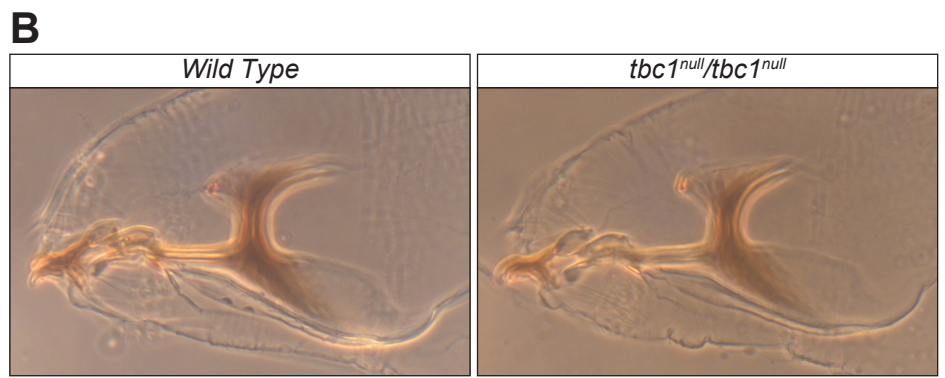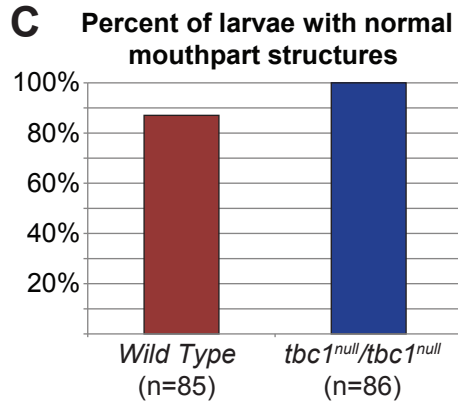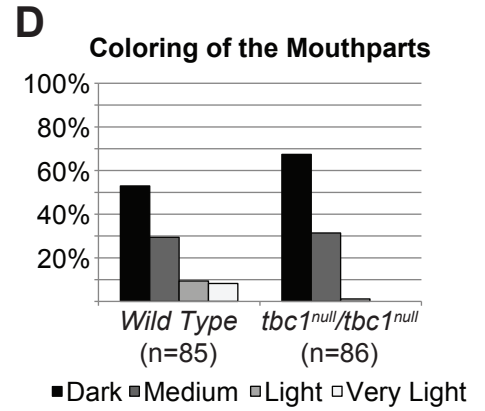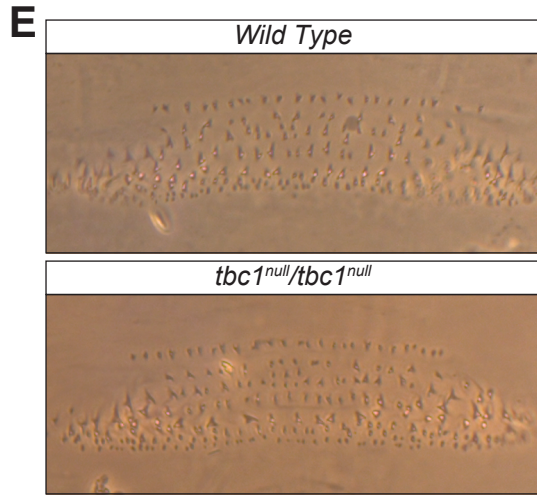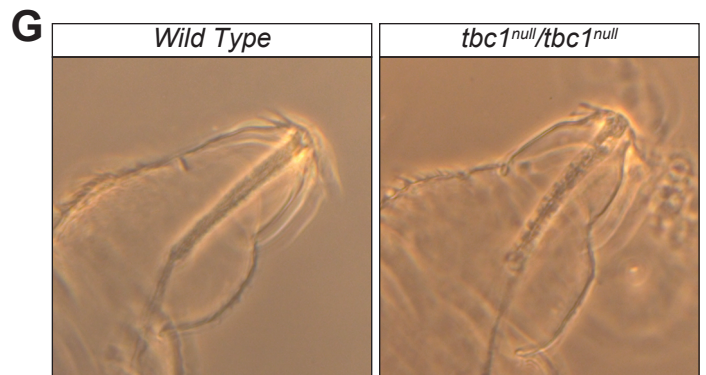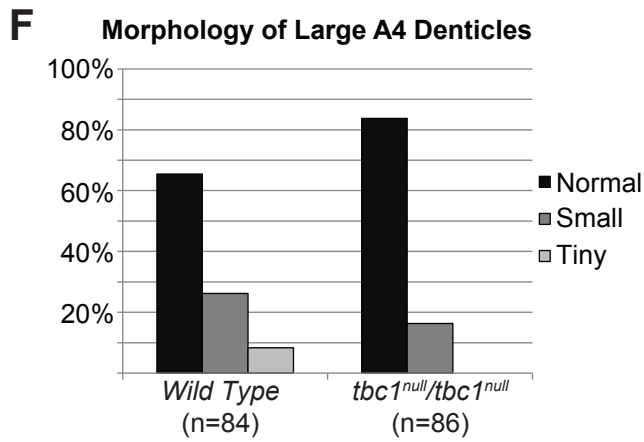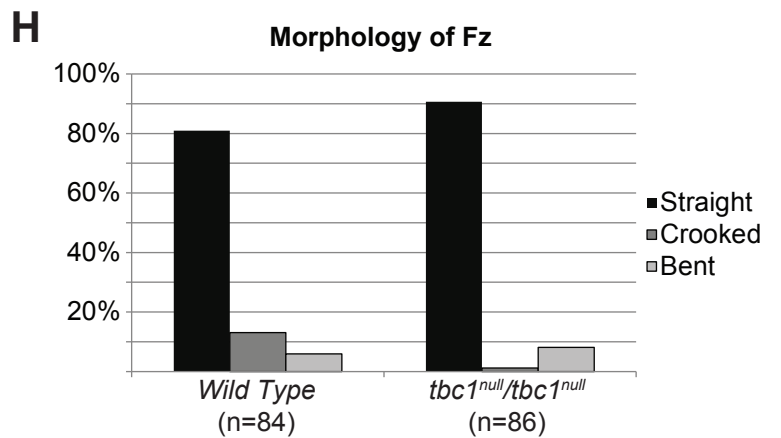

Supplement: Supplementary file 4 — Figure S4. Loss of tbc1 does not affect larval cuticle morphology. (A) 100X dark field images of lateral views of cuticle preparations from wild type and tbc1 null homozygotes. (B) 400X phase images of the cuticle preparations showing the mouthparts of a wild type and tbc1 null homozygotes larva. (C,D) Quantification of irregularities and pigmentation defects found in wild type and tbc1 null mouthparts. (E) 400X images of the A4 denticle belts from wild type and tbc1 null larvae. (F) Quantification of largest denticle size in WT and tbc1 null larvae. (G) 400X images of filzkörper (FK) from wild type and tbc1 null larvae. (H) Quantification of structural FK irregularities in wild type and tbc1 null larvae. (PDF 8359 kb) [file 12860_2019_198_MOESM4_ESM.pdf]
