# Supplementary material for: Role of tbc1 in Drosophila embryonic salivary glands
Source: BMC Mol Cell Biol. 2019 Jun 26;20:19. doi: 10.1186/s12860-019-0198-z (PMC6595604; doi:10.1186/s12860-019-0198-z)
Supplement: Supplementary file 5 — Figure S5. Maternal loss of tbc1 increases the percentage of progeny that completely fail to produce cuticle Wild-type (Oregon R) or tbc1 homozygous females were crossed to either wild-type or tbc1 homozygous males. Between 85 and 92% of progeny of WT females developed into first instar larvae, whereas only between 65 and 75% of progeny of tbc1 null females developed into first instar larvae. (PDF 116 kb) [file 12860_2019_198_MOESM5_ESM.pdf]

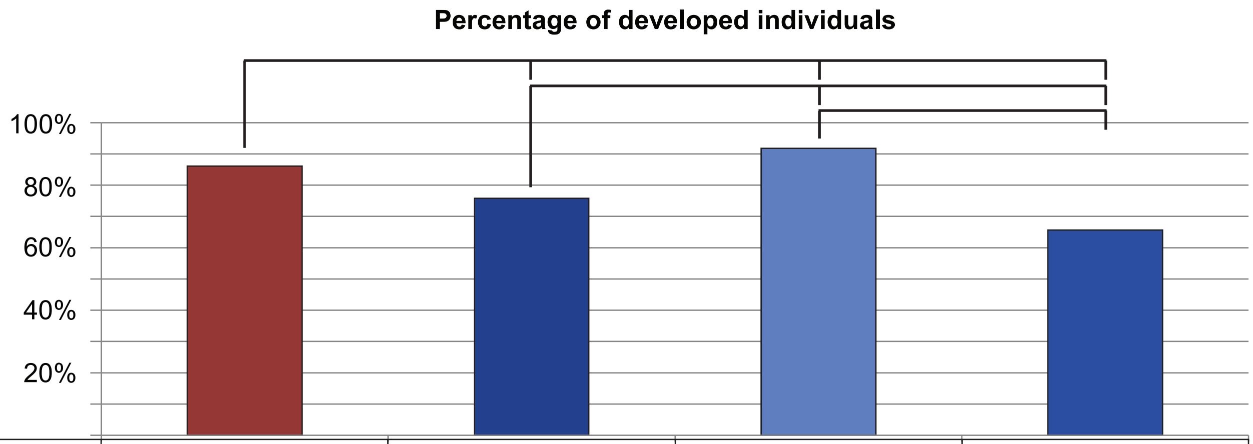

Maternal Genotype	*Wild Type*	$\frac{tbc1^{null}}{tbc1^{null}}$	*Wild Type*	$\frac{tbc1^{null}}{tbc1^{null}}$
Paternal Genotype	*Wild Type*	$\frac{tbc1^{null}}{tbc1^{null}}$	$\frac{tbc1^{null}}{tbc1^{null}}$	*Wild Type*
n	1944	1046	183	211
