# Supplementary material for: Role of tbc1 in Drosophila embryonic salivary glands
Source: BMC Mol Cell Biol. 2019 Jun 26;20:19. doi: 10.1186/s12860-019-0198-z (PMC6595604; doi:10.1186/s12860-019-0198-z)
Supplement: Supplementary file 6 — Table S1. Rab-YFP lines used. Table S2. Primers used. Table S3 Antibodies used (DOCX 98 kb) [file 12860_2019_198_MOESM6_ESM.docx]

**Supplemental Table 1 Rab-YFP lines used**

| **Rab** | **Stock Number** | **Stock Genotype** | **Identifier** | **Citation** |
| --- | --- | --- | --- | --- |
| Rab10 | 9789 | y1 w*; P{UASp-YFP.Rab10}21 | RRID:BDSC_9789 | [26] |
| Rab14 | 9793 | y^1^ w^*^; P{UASp-YFP.Rab14}CG9967^5L^ | RRID:BDSC_9793 | [26] |
| Rab18 | 9796 | y1 w*; P{UAST-YFP.Rab18}CG977501 | RRID:BDSC_9796 | [26] |
| Rab19 | 24150 | y1 w*; P{UAST-YFP.Rab19}Hr3902 | RRID:BDSC_24150 | [26] |
| Rab21 | 23242 | y1 w*; P{UAST-YFP.Rab21}pog04 | RRID:BDSC_23242 | [26] |
| Rab23 | 9802 | y[1] w[*]; ; P{w[+mC]=UASp-YFP.Rab23}01 | RRID:BDSC_9802 | [26] |
| Rab26 | 23245 | y[1] w[*]; P{w[+mC]=UAST-YFP.Rab26}05/CyO | RRID:BDSC_23245 | [26] |
| Rab30 | 9812 | y1 w*; P{UASp-YFP.Rab30}Cdk410 | RRID:BDSC_9812 | [26] |
| Rab35 | 9821 | y^1^ w*; P{UASp-YFP.Rab35}15 | RRID:BDSC_9821 | [26] |
| Rab4 | 23269 | y1 w*; P{UASp-YFP.Rab4}09 | RRID:BDSC_23269 | [26] |
| Rab5 | 24616 | y^1^ w^*^; P{UASp-YFP.Rab5}02 | RRID:BDSC_24616 | [26] |
| Rab6 | 23251 | y^1^ w^*^; P{UAST-YFP.Rab6}CG10082^01^/CyO | RRID:BDSC_23251 | [26] |
| Rab7 | 42705 | w*; P{UAS-Rab7.GFP}2 | RRID:BDSC_42705 | [44] |
| Rab8 | 9782 | y1 w*; P{UASp-YFP.Rab8}45 | RRID:BDSC_9782 | [26] |
| Rab9 | 9784 | y1 w*; P{UASp-YFP.Rab9}22 | RRID:BDSC_9784 | [26] |
| RabX1 | 9840 | y1 w*; P{UASp-YFP.RabX1}12 | RRID:BDSC_9840 | [26] |
| RabX4 | 9851 | y1 w*; P{UASp-YFP.RabX4}how19 | RRID:BDSC_9851 | [26] |

**Supplemental Table 2 Primers used**

| **Tbc1 primers** | | |
| --- | --- | --- |
| Primer Name | Primer Sequence | Use of primer |
| CG4552_5L | GGGCGGCCGCCGACAAATAACACTTGTGGAAGTC | 5' HR Region for *tbc1*; forward |
| CG4552_5R | GGGGTACCTAAGGTTATTCAGCGGGATCTAAG | 5' HR Region for *tbc1*; reverse |
| CG4552_3L | GGGGCGCGCCTCATCACGTTCAAGTACGGATTC | 3' HR Region for *tbc1*; forward |
| CG4552_3R | GGCGTACGGTAGGTTCCCGTGTGCTTG | 3' HR Region for *tbc1*; reverse |
| CG4552 KO | gggttgggatgggttggggc | Diagnostics: Upstream of HR for *tbc1* KO; forward |
| DJ015 | cacacacactcacaaaggagg | Diagnostics: Downstream of HR for *tbc1* KO; reverse |
| DJ066 | cgcgcggcagccatatg ACAGAGTTTCCTGTGCCGGATG | Tbc1 cloning into pET15b protein expression; forward |
| DJ050 | ccggatcctcgagcatatg CTACTTGGCGTCGTCAAGCACC | Tbc1 cloning into pET15b protein expression; reverse |
| DJ042 | cacc ATGGAGGAGAATATGTGGATC | Tbc1 cloning into pENTRD vector; forward |
| DJ043 | CTACTTGGCGTCGTCAAGCACC | Tbc1 cloning into pENTRD vector; untagged, reverse |
| DJ044 | CTTGGCGTCGTCAAGCACCTGCA | Tbc1 cloning into pENTRD vector; forward |
| **White primers** | | |
| Primer Name | Primer Sequence | Use of primer |
| PW25Primer2 | ACTGTGCGACAGAGTGAGAG | In w+ gene in pw25 construct; reverse |
| PW25Primer3 | GGTCGACTCTAGAGGATCAT | In w+ gene in pw25 construct; forward |

**Supplemental Table 3 Antibodies used**

| **Primary Antibodies** | | | | |
| --- | --- | --- | --- | --- |
| **Antibody** | **Species** | **Concentration** | **Source** | **RRID** |
| Crb | Mouse | 1:40 | DSHB | AB_528181 |
| GFP | Rabbit | 1:10,000 | Life Technologies | A11122 |
| en | Rabbit | 1:100 | [27] |  |
| SG2 | Rabbit | 1:8,000 | [9] |  |
| GFP | Mouse | 1:2,000 | Molecular Probes |  |
| β-Gal | Mouse | 1:1,000 | Promega |  |
| Tbc1 | Rat | 1:10 | This work |  |
| GM130 | Rabbit | 1:100 | Abcam |  |
| Golgin-245 | Goat | 1:400 | DSHB; [45] | AB_2569587 |
| Rab11 | Rabbit | 1:500 | Yim and Andrew (unpublished) |  |
| DCSP1 | Mouse | 1:100 | DSHB | AB_2307345 |

| **Secondary Antibodies** | | | | |
| --- | --- | --- | --- | --- |
| **Antibody** | **Species** | **Conjugate** | **Concentration** | **Source** |
| Rabbit | Goat | Biotin | 1:500 | Invitrogen Molecular Probes |
| Mouse | Goat | 488 | 1:500 | Invitrogen Molecular Probes |
| Rabbit | Goat | 568 | 1:500 | Invitrogen Molecular Probes |
| Guinea Pig | Goat | 647 | 1:500 | Invitrogen Molecular Probes |
| Mouse | Goat | Biotin | 1:500 | Invitrogen Molecular Probes |
| Rat | Donkey | 647 | 1:500 | Invitrogen Molecular Probes |
| Rabbit | Donkey | 568 | 1:500 | Invitrogen Molecular Probes |
| Goat | Donkey | 488 | 1:500 | Invitrogen Molecular Probes |
| Rabbit | Goat | 488 | 1:500 | Invitrogen Molecular Probes |
| Mouse | Goat | 568 | 1:500 | Invitrogen Molecular Probes |
